# Both Vitamin D Supplementation and HIIT Boost Muscle VDR Expression, Which May Underlie Benefits for Frailty

**DOI:** 10.1093/geroni/igab046.2550

**Published:** 2021-12-17

**Authors:** Ken Seldeen, Ramkumar Thiyagarajan, Merced Leiker, Reem Berman, Yonas Redae, Carleara Weiss, Lee Chaves, Bruce Troen

**Affiliations:** University at Buffalo, Buffalo, New York, United States

## Abstract

Frailty is a condition of poor response to stressors that increases susceptibility to adverse outcomes - including disability and death. Declining physical function is an important hallmark of frailty, and we previously published that long term vitamin D insufficiency from young to middle-age leads to declines in endurance and gait disturbances. Furthermore, we report that aged mice (24-months) made vitamin D insufficient for 4 months exhibit increased frailty, whereas those made hyper-sufficient do not. Exercise, including short session high intensity interval training (HIIT - 10 minutes/3x-week), also reverses frailty in aged mice. Here we investigate the impacts of aging, vitamin D, and exercise on underlying muscle quality and muscle stem cell activity. Our preliminary data reveal muscle vitamin D receptor (VDR) expression is lower in aged mice (24-28 months) relative to young mice (6 months). Yet HIIT, either one hour after a single session or following 6 weeks, increases VDR expression. HIIT also increases myonuclear accretion in muscle fibers – an indicator of in vivo stem cell activity – and stimulates progenitor cells proliferation ex vivo. Likewise, we observe that vitamin D hyper supplementation alone also increases muscle VDR expression and the number of satellite cells. These data indicate that both vitamin D supplementation and HIIT independently enhance VDR expression in skeletal muscle with associated greater satellite and muscle progenitor cell activity. These data critically link vitamin D physiology and HIIT in muscle, and thus provide a mechanistic basis for their benefits for muscle quality, function, and health during aging.

